# Differences in predictive factors for post-traumatic stress disorder encompassing partial PTSD and full PTSD: a cross-sectional study among individuals exposed to the November 13, 2015 Paris attacks

**DOI:** 10.3389/fpsyt.2024.1351695

**Published:** 2024-03-20

**Authors:** Benoit Berthail, Marion Trousselard, Gregory Lecouvey, Barbara Le Roy, Florence Fraisse, Denis Peschanski, Francis Eustache, Pierre Gagnepain, Jacques Dayan

**Affiliations:** ^1^ French Military Health Service Academy, Ecole du Val de Grace, 1 Place Alphonse Laveran, Paris, France; ^2^ Neuropsychology and Imaging of Human Memory (NIMH) Research Unit, GIP Cyceron, INSERM U1077, Caen University Hospital, PSL, EPHE, Caen University, Caen, France; ^3^ Stress Neurophysiology Unit, French Armed Forces Biomedical Research Institute, Brétigny-sur-Orge, France; ^4^ University of Lorraine, Inserm, INSPIIRE UMR 1319, F-54000, Nancy, France; ^5^ Paris I Pantheon Sorbonne University, HESAM University , EHESS, CNRS, UMR8209, Paris, France; ^6^ Child and Adolescent Psychiatry University Hospital Pole, Guillaume Régnier Hospital Center, Rennes 1 University, 35700 Rennes, France

**Keywords:** post-traumatic stress disorder, partial post-traumatic stress disorder, terrorist attack, predictive factors, Paris attacks

## Abstract

**Background:**

When faced with a surge of physically injured individuals, especially following a traumatic event like an attack, frontline practitioners prioritize early triage. Detecting potential psychological injuries soon after such events remains challenging. Some individuals might develop post-traumatic stress disorder (PTSD) according to DSM-V criteria. Others may exhibit PTSD symptoms without meeting full diagnostic criteria, termed partial or sub-syndromal PTSD, a less-explored area in literature. This study aims to identify predictive factors for both full and partial PTSD.

**Method:**

In a cohort of victims of the 2015 Paris attacks, multinomial logistic regressions explored predictive factors for partial or full PTSD status 8 to 18 months post-attacks. Analyses considered pre, peri, and posttraumatic factors chosen from literature review and univariate analysis within each group.

**Results:**

Within the cohort, 50 individuals showed no signs of PTSD, 35 experienced partial PTSD, and 30 presented with full PTSD. After logistic regression, risk factors associated with full PTSD included a history of trauma (OR = 1.30, CI [1.02-1.66], p < 0.05), the intensity of peri-traumatic physical reactions (OR = 1.22, CI [1.09-1.36], p < 0.001), the difficulties in suppressing intrusive thoughts (OR = 1.11, CI [1.02-1.21], p < 0.013). Only the intensity of peri-traumatic physical reactions emerged as a risk factor for partial PTSD (OR = 1.13, [CI 1.02-1.24], p < 0.001).

**Discussion:**

This study revealed that a history of trauma, the intensity of peri-traumatic physical reactions (e.g., tachycardia, trembling, flushes, numbness.), and the difficulties in suppressing intrusive thoughts constitute risk factors for the development of full PTSD. Moreover, the study identified that only the intensity of peri-traumatic physical reactions emerged as a risk factor for partial PTSD. These findings seem to underscore the significance of peri-traumatic experiences in influencing the development of post-traumatic stress symptoms.

**Conclusion:**

This study emphasizes the significance of examining peri-traumatic reactions in PTSD development, suggesting its potential as a straightforward screening tool for post-traumatic stress disorder. It also underscores the influence of prior traumatic experiences, before *de novo* traumatization, in shaping vulnerability to PTSD and illuminates the crucial role of compromised control of intrusive thoughts that could perpetuate PTSD.

## Highlights

Our study explores the predictive factors of post-traumatic stress disorder. Trauma history, intensity of peri-traumatic physical reactions and the difficulties in suppressing intrusive thoughts are risk factors for post-traumatic stress disorder.

## 1 Introduction

Current literature abundantly highlights the existence of important variabilities in the risk of developing Posttraumatic Stress Disorder (PTSD) after a traumatic event.

The PTSD assessment is enhanced by the DSM5 (1), a reference guide used by mental health professionals to diagnose mental disorders. It provides specific criteria for each disorder, including PTSD, based on observed symptoms in individuals after experiencing a traumatic event. DSM5 criteria for PTSD include symptoms grouped into four categories: Criteria A (exposure to a traumatic event), Criteria B (intrusive reactions), Criteria C (avoidance), Criteria D (negative alterations in cognition or mood), Criteria E (alterations in reactivity), and Criteria F, G, and H (duration and clinical disruption).

The literature on risk factors for PTSD is vast, encompassing pre-, peri-and post-trauma variables independent of acute stress disorder (ASD) (2, 3). Pre-traumatic risk factors are elements present before a traumatic event that can increase an individual’s vulnerability to developing mental health issues following trauma. It includes an individual’s psychiatric history, cumulative traumatic events, and level of preparedness (4), being female (5, 6), disruption of the individual’s concept of reality (7, 8), vulnerable personality (9, 10). Peri-traumatic risk factors refer to elements occurring during or immediately after a traumatic event and includes peritraumatic dissociation (11, 12), intensity of the peritraumatic response (13, 14), as indicated by increased autonomic nervous system activity and fight or flight hormones (15, 16), duration of exposure and proximity to danger and death (11, 17), physical injury (18, 19), intentional nature of the attack (20). Post-traumatic risk factors are elements that arise after the traumatic event and can prolong or exacerbate its negative effects. It involves lack of social support (14), experience of the death of a friend or loved one, financial hardship, and inability to return to work (21).

Traumatic exposure can elicit diverse clinical responses, potentially giving rise to at least partial post-traumatic stress disorder (PTSD) or full PTSD (22). Recognizing the significance of individuals displaying some, but not all, PTSD symptoms following a traumatic event has gained consensus among researchers (22–24). For instance, a longitudinal study focusing on police officers involved in the World Trade Center bombing intervention revealed a gradual increase in reported PTSD symptoms over time (22).

The concept of ‘partial’ PTSD, initially applied to describe Vietnam veterans (25), lacks consistent criteria across studies (26). Some define it based on the presence of two out of the three DSM5 criteria (B, C, and D) (27, 28), while others refer to it as ‘subthreshold’ PTSD (29, 30). In this study, partial PTSD was defined as meeting DSM5 criteria A, B, F, G, and H, with traumatic intrusions and enduring clinical suffering lasting over one month.

Despite the challenge of a clear and universal definition, studies indicate that the true impact of traumatic events on populations with PTSD symptoms is often underestimated (31). Individuals with partial PTSD exhibit higher associated psychiatric comorbidities (23, 29, 31) and experience social dysfunction (32). Interestingly, those with partial PTSD, although less clinically impaired than those with full PTSD, demonstrate functional impairment related to their symptoms and seek care with comparable frequency (24).

However, given the wide range of risk factors identified in the literature, it is challenging to identify factors that could determine predictive models of individual risk for post-traumatic stress after trauma. Accurately identifying the risk factors for PTSD is crucial for medical teams who coordinate the management of victims of traumatic events (33). Despite medical and psychological interventions, more than 20% of individuals exposed to trauma do not respond to treatment, and approximately 40% of those who do recover experience a relapse within a year (34).

The intentional nature of events like terrorism, likely intensified psychological trauma and its persistence over time compared to accidental events (35).

The Paris attacks, a series of terrorist attacks that occurred on November 13, 2015, had a profound impact, causing significant trauma. They targeted diverse groups in multiple public spaces, collectively threatening daily life and received extensive media coverage. The attacks involved kamikaze bombs around the “Stade de France”, shootings and bombings in different locations in the 10th and 11th arrondissements of Paris, and an attack on the Bataclan theatre, resulting in 130 deaths and 354 injuries (36). Set in Paris, a cultural symbol, the attacks influenced social fabric, politics, and security. These factors, alongside the attacks’ sudden brutality and the intentional nature of events like terrorism, likely intensified psychological trauma, increasing the risk of PTSD and related disorders. Grasping this context is essential for studying PTSD risk factors.

Thus, the main objective of this work is to identify risk factors for PTSD, including partial PTSD, in order to predict post-exposure clinic and improve medical follow-up, by proposing that prior traumatic experiences, peritraumatic physical reactions, peritraumatic dissociation, and difficulties in suppressing intrusive thoughts are risk factors for full and partial PTSD. This study focuses on identifying risk factors, rather than vulnerability factors. The study considers pre-trauma, peri-trauma, and post-trauma factors that have been most studied in the literature on PTSD risk factors.

## 2 Materials

### 2.1 Participants and procedures

This monocentric cross-sectional analytical study is a component of the REMEMBER (REsilience and Modification of brain control network following novEMBER 13) biomedical research project, which received prior approval from the Nord Ouest III Personal Protection Committee (12/2016; ID RCB: 2016-A00661-50). Written consent was obtained from all subjects before participation.

It consists of a neuropsychological analysis component, integrated into this work, and a functional imaging analysis. It is an ancillary study to the sociological study “Étude 1000,” aiming to analyze the testimonies of 1000 volunteers across 4 exposed and non-exposed groups. The first group (n=360) comprises individuals exposed to attacks according to DSM-5 Criterion A (survivors, witnesses, bereaved relatives, first responders, directly present at any of the attack sites on the evening of November 13, 2015 (including both spontaneous bystanders and professional responders such as emergency services, law enforcement, etc.), or in close proximity to a victim injured or killed during or as a result of the attacks, or present near the site of the assault on November 18, 2015, in Saint-Denis), recruited through victim associations and volunteer calls (37).

Within this group, individuals were invited to a neuropsychological study, subject to restrictions, to form the REMEMBER study analysis group. However, only 120 responded positively, primarily due to the distance to the study location in Caen (approximately 200 km from Paris) and reluctance towards undergoing fMRI examinations. Out of 120 subjects, 115 were included in our study analysis. Data from 5 non-exposed participants were excluded: one couldn’t continue, one wasn’t truly exposed, one didn’t meet the inclusion criterion, and two exhibited re-experiencing symptoms without other categories (including functional significance, Criterion G).

All participants met inclusion criteria: aged 18-60 regardless of gender, right-handed, French-speaking, with BMI <35 kg/m2, individuals comprising the primary exposed group of the “Étude 1000,” enrolled in a social security scheme, have provided written informed consent. Exclusion criteria included being pregnant or planning pregnancy, individuals detained by judicial or administrative order, individuals residing in health or social institutions for reasons unrelated to research, participants currently excluded from another research project, history of severe psychiatric conditions such as psychotic disorders, bipolar disorders, obsessive-compulsive disorders, and/or addictive disorders (pre-existing prior to November 13, 2015, excluding tobacco addiction), history of neurological disorders (stroke, epilepsy, head trauma resulting in loss of consciousness for over one hour), use of medication known to affect cognitive and/or cerebral function, and conditions precluding MRI scanning (e.g., claustrophobia, metal implants). Subject inclusion and neuropsychological testing took place between June 13, 2016, and June 7, 2017, 8 to 18 months after the attacks (average one year). Semi-structured interviews and self-reported questionnaires were used to determine the presence of PTSD and to assess protective or risk factors for its development. Measures of post-traumatic stress disorder, pre-traumatic, peri-traumatic, and post-traumatic factors were carried out using questionnaires validated in French and English and chosen for their psychometric and clinical qualities.

### 2.2 Measurement of PTSD

The Structured Clinical Interview for DSM5 (SCID) (1) was used to diagnose possible disorders related to exposure to the attacks. Any individual meeting criterion A was classified as having full PTSD if they completely met DSM5 specifications, placing them within the *exposed group with full PTSD*.

Individuals meeting DSM5 criteria A, B, F, G, and H, with traumatic intrusions and enduring clinical suffering lasting over one month, were classified within the *exposed group with partial PTSD*. Participants failing to meet criteria B and/or G were categorized as not experiencing either full or partial PTSD, thereby belonging to the *exposed group without PTSD*.

### 2.3 Measurement of pre-traumatic factors

Socio-demographic data, including the subject’s marital status, professional situation, and level of education were evaluated by a dedicated questionnaire. A childhood questionnaire was used to identify childhood history. The life events checklist for DSM5 was used to determine anterior exposure of traumatic events (38). Finally, the MINI test (Mini International Neuropsychiatric Interview; 39), which is a structured diagnostic interview, was used to evaluate the main psychiatric disorders of the DSM5 during lifetime in a standardized manner.

### 2.4 Measurement of peri-traumatic factors

Among the 115 participants, the level of exposure was assessed by DSM5 criterion A, which determines whether the subject experienced the trauma directly (criterion A1, *n=78*), witnessed the event experienced by others (criterion A2, *n=14*), was a close friend or close family member of someone who experienced the trauma (criterion A3, *n=6*), or was repeatedly exposed to the distressing details of the traumatic event, such as first responders (criterion A4, *n=17*). Initial reactions were assessed via the Initial Subjective Reaction Physical Scale of the Potential Stressful Events Interview (40) and Initial Subjective Reaction Emotional Scale of the Potential Stressful Events Interview (40). The existence of a peri-traumatic dissociative syndrome was investigated using the Peritraumatic Dissociative Experiences Questionnaire-Self-Report Version (41).

### 2.5 Measurement of post-traumatic factors

The nature of the reaction, particularly coping processes since the attacks was assessed by using the Brief COPE Inventory (42). The difficulties in suppressing intrusive thoughts has employed the White Bear Suppression Inventory (WBSI, 43). The WBSI concurrently measures the tendency to engage in thought suppression and the frequency of associated intrusive thoughts. Notably, increased efforts to suppress thoughts is often associated with heightened intrusive thoughts (44). This phenomenon has been proposed to reflect a consequence of a compromised inhibitory control system regulating memory activity (45, 46), that could elucidate why some individuals with PTSD encounter challenges in suppressing intrusive thoughts and tend to endorse a higher number of items on the WBSI (47). Depression was measured using the Beck Depression Inventory (48). Anxiety was assessed by the State-Trait Anxiety Inventory, Form Y (STAI-Y; 49). Self-report questionnaires were used to determine the pattern of alcohol use following the trauma. Finally, other vulnerability factors such as social-economic and social-professional deficits were sought by the social support questionnaire.

### 2.6 Statistical analyses

**Figure 1:**
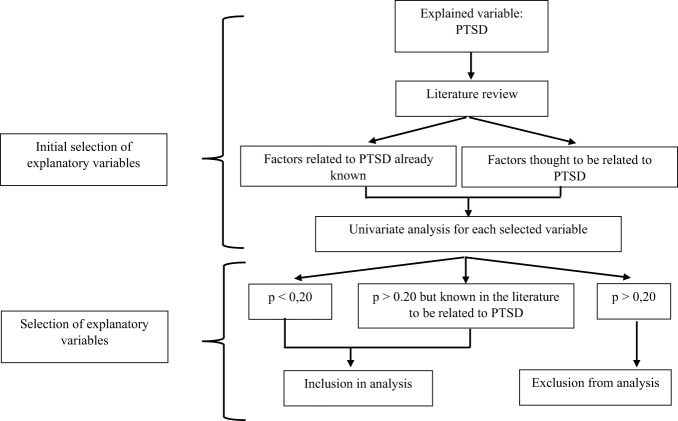
Diagram explaining the process of selecting the variables to be included in the multiple logistic regression model.

The statistical methods used to identify explanatory factors for the development of PTSD in trauma-exposed victims included both univariate multinomial logistic regression and multivariate multinomial logistic regression analyses. The group without PTSD was used as the reference group in the analyses. Typically, variables with a p-significance level of less than 0.20 in the univariate analysis were included in the initial multiple logistic regression model (50). This threshold allows for the consideration of possible confounding factors. Additionally, variables that are known in the literature to be associated with pathology but did not reach the significance level of 0.20 in the univariate analysis were also included in the initial model. Finally, in cases of redundancy between variables, only the most significant variable was included in the model (50), (**Figure 1**).

When aiming to develop an explanatory model, certain procedures can be implemented to select the variables to be included. The objective is to select the model that provides the maximum amount of information on the variable to be explained (Y) from the smallest possible number of explanatory variables (Xi) while adhering to the principle of parsimony (using the fewest possible explanatory variables to explain a phenomenon) (50). To facilitate model estimations and limit problems related to missing data, individuals with missing data were removed from the analysis (13% of the sample).

It is also important to ensure that there are enough events (e.g., patients with PTSD) compared to the number of explanatory variables (Xi). A general rule is to have at least ten times more events than explanatory variables included in the logistic regression model (50).

To retain as many explanatory variables as possible without decreasing power, the selected variables were studied in three independent time blocks: pre-trauma, peri-trauma, and post-trauma. Multiple logistic regression was performed on each block to obtain an explanatory model for the impact of the predictors on the presence or absence of PTSD.

These analyses were performed on R via the glm-package.

## 3 Results

### 3.1 Participants

Of the 115 included subjects, 50 participants (43.5% of the total sample) had no PTSD symptoms, and 65 (56.5% of the total sample) exhibited PTSD symptoms. The latter group can be divided into two subgroups according to their PTSD status: a group of 35 participants (30.4% of the total sample) with partial PTSD, and a group of 30 participants (26.1% of the total sample) with full PTSD.

56 participants were women (48.7%) and 59 were men (51.3%). The average age of the study population was 36.4 years (standard deviation: 7.57). Furthermore, 93% were educated to high school level (the French *baccalaureate*), and 40% had received a further five years (or more) of education. Finally, 53% were married or in a common-law relationship, and 83.5% were in employment. **Table 1** provides an overview of the descriptive statistics for the study population.

**Table 1:** Socio-demographic statistics for the study population as a function of PTSD group (no PTSD, partial PTSD, full PTSD).

Characteristic	No PTSD, N = 50^1^	Partial PTSD, N = 35^1^	Full PTSD, N = 30^1^
Sex			
*Men*	30 (60%)	16 (46%)	13 (43%)
*Women*	20 (40%)	19 (54%)	17 (57%)
Age	34 (31,41)	36 (30,43)	36 (31,40)
Marital status			
*Single*	16 (32%)	15 (43%)	15 (50%)
*In a relationship*	15 (30%)	8 (23%)	8 (27%)
*Married or civil partnership*	14 (28%)	9 (26%)	7 (23%)
*Separated, divorced or widower*	5 (10%)	3 (8.6%)	0 (0%)
Professional status			
*No activity*	8 (16%)	4 (11%)	7 (23%)
*In activity*	42 (84%)	31 (89%)	23 (77%)
Education level			
*<Bac*	3 (6.0%)	3 (8.8%)	1 (3.3%)
*Bac – Bac+5*	23 (46%)	18 (53%)	20 (66.7%)
*> Bac+5*	24 (48%)	13 (38%)	9 (30%)
*Unknown*	0 (0%)	1 (0.2%)	0 (0%)

^1^n (%); Median (IQR).

### 3.2 Pre-traumatic factors

**Table 2** presents the results of the univariate analysis performed on the pre-trauma variables selected for the multiple logistic regression analysis, as described in the methodology section. Psychiatric and trauma history were included in the multiple logistic regression analysis as their p-value was less than 0.20. Age and sex were also included in the multivariate analysis, as they are known risk factors according to the literature, regardless of their p-values (p=0.8 and p=0.3, respectively).

**Table 2:** Results of univariate analysis of pre-trauma factors.

Characteristic	No PTSD, N = 44^1^	Partial PTSD, N = 31^1^	Full PTSD,N = 25^1^	p-value^2^
**Sex**				0.3
* Men*	27 (61%)	15 (48%)	11 (44%)	
* Women*	17 (39%)	16 (52%)	14 (56%)	
**Marital status**				0.3
* Single*	14 (32%)	14 (45%)	15 (60%)	
* In a relationship*	14 (32%)	6 (19%)	5 (20%)	
* Married or civil partnership*	13 (30%)	8 (26%)	5 (20%)	
* Separated, divorced or widower*	3 (6.8%)	3 (9.7%)	0 (0%)	
**Professional status**				0.6
* No activity*	7 (16%)	4 (13%)	6 (24%)	
* In activity*	37 (84%)	27 (87%)	19 (76%)	
**Education level**				0.6
* <Bac*	3 (6.8%)	3 (9.7%)	1 (4.0%)	
* Bac – Bac+5*	20 (45%)	17 (55%)	16 (64%)	
* > Bac+5*	21 (48%)	11 (35%)	8 (32%)	
**Psychiatric history**				**0.090**
* Absence*	32 (73%)	17 (55%)	12 (48%)	
* Presence*	12 (27%)	14 (45%)	13 (52%)	
**Age**	34 (31, 40)	36 (30, 44)	36 (30, 40)	0.8
**Childhood history**	2.00 (1.00, 4.00)	2.00 (1.00, 3.50)	3.00 (2.00, 5.00)	0.3
**Trauma history**	2.00 (0.75, 3.00)	1.00 (0.00, 3.00)	3.00 (1.00, 4.00)	**0.031**

^1^n (%); Median (IQR).

^2^Pearson’s Chi-squared test; Fisher’s exact test; Kruskal-Wallis rank sum test.

The p-values highlighted in bold are the values that were included in the multivariate logistic regression analysis because they were p < 0.20.

After conducting the multivariate analysis on the pre-trauma factors, it was found that trauma history emerged as a significant risk factor solely for full PTSD (OR=1.30, 95% CI [1.02-1.66], p<0.05). In contrast, neither psychiatric history, gender nor age were identified as significant factors for either full or partial PTSD (**Table 3**).

**Table 3:** Association between potential pre-traumatic predictors and presence of full and partial PTSD in a multivariate logistic regression (N =99).

Characteristic	OR*^1^*	95% CI*^1^*	p-value
Partial PTSD			
Trauma history	0.96	0.75, 1.25	**0.038**
Sex			0.20
* Men*	–	–	
* Women*	2.01	0.75, 5.34	
Psychiatric history			0.094
* Absence*	–	–	
* Presence*	2.20	0.81, 5.97	
Age	1.03	0.97, 1.10	0.66
Full PTSD			
Trauma history	1.30	1.02, 1.66	**0.038**
Sex			0.20
* Men*	–	–	
* Women*	2.37	0.81, 6.97	
Psychiatric history			0.094
* Absence*	–	–	
* Presence*	3.02	1.02, 8.98	
Age	1.01	0.94, 1.09	0.66

^1^OR, Odds Ratio; CI, Confidence Interval.

The p-values highlighted in bold are those that emerged as significant following multivariate logistic regression analysis as they were below p < 0.05.

### 3.3 Peri-traumatic factors

**Table 4** presents the results of the univariate analysis of the peri-trauma variables selected for the multiple logistic regression analysis, as described in the methodology. Specifically, degrees of exposure (direct exposure and repeated exposure to painful details), intensity of physical and emotional reactions, and peritraumatic dissociation were included in the multiple logistic regression (p < 0.20).

**Table 4:** Results of univariate analysis of peri-trauma factors.

Characteristic	No PTSD, N = 50^1^	Partial PTSD, N = 35^1^	Full PTSD, N = 30^1^	p-value^2^
**Direct exposure**				**0.004**
* Absent*	24 (48%)	5 (14%)	8 (27%)	
* Present*	26 (52%)	30 (86%)	22 (73%)	
**Direct witness**				0.8
* Absent*	43 (86%)	32 (91%)	26 (87%)	
* Present*	7 (14%)	3 (8.6%)	4 (13%)	
**Traumatic event involving a close relative**				0.5
* Absent*	48 (96%)	34 (97%)	27 (90%)	
* Present*	2 (4.0%)	1 (2.9%)	3 (10%)	
**Repeated exposure to painful details**				**<0.001**
* Absent*	35 (70%)	34 (97%)	29 (97%)	
* Present*	15 (30%)	1 (2.9%)	1 (3.3%)	
**Initial physical reactions**	14 (12, 19)	21 (15, 26)	25 (21, 33)	**<0.001**
**Initial emotional reactions**	32 (25, 38)	37 (32, 41)	40 (36, 47)	**<0.001**
**Peritraumatic dissociation**	33 (20, 45)	34 (25, 54)	45 (32, 52)	0.089

^1^n (%); Median (IQR).

^2^Pearson’s Chi-squared test; Fisher’s exact test; Kruskal-Wallis rank sum test.

The p-values highlighted in bold are the values that were included in the multivariate logistic regression analysis because they were p < 0.20.

Subsequently, a multivariate logistic regression was performed on these variables, and the results are presented in **Table 5**. The analysis revealed that only the intensity of peri-traumatic physical reactions was a significant risk factor for both partial PTSD (OR = 1.13, 95% CI [1.02-1.24], p < 0.001) and full PTSD (OR = 1.22, 95% CI [1.09-1.36], p < 0.001).

**Table 5:** Association between potential peri-traumatic predictors and presence of full and partial PTSD in a multivariate logistic regression (N =99).

Characteristic	OR*^1^*	95% CI*^1^*	p-value
Partial PTSD			
Direct exposure			0.23
* Absent*	–	–	
* Present*	2.94	0.76, 11.4	
Repeated exposure to painful details			0.32
* Absent*	–	–	
* Present*	0.24	0.02, 2.74	
Initial emotional reactions	0.99	0.92, 1.07	0.94
Initial physical reactions	1.13	1.02, 1.24	**<0.001**
Peritraumatic dissociation	1.00	0.97, 1.03	0.99
Full PTSD			
Direct exposure			0.23
* Absent*	–	–	
* Present*	1.29	0.33, 4.96	
Repeated exposure to painful details			0.32
* Absent*	–	–	
* Present*	0.26	0.02, 3.06	
Initial emotional reactions	1.01	0.92, 1.10	0.94
Initial physical reactions	1.22	1.09, 1.36	**<0.001**
Peritraumatic dissociation	1.00	0.97, 1.04	0.99

^1^OR, Odds Ratio; CI, Confidence Interval.

The p-values highlighted in bold are those that emerged as significant following multivariate logistic regression analysis as they were below p < 0.05.

### 3.4 Post-traumatic factors

**Table 6** presents the results of the univariate analysis of the post-trauma variables selected for multiple logistic regression, as described in the methodology. Variables with a p-value <0.20 in the univariate analysis were included in the multivariate analysis.

**Table 6:** Results of univariate analysis of post-traumatic factors.

Characteristic	No PTSD, N = 48^1^	Partial PTSD, N =32^1^	Full PTSD, N = 26^1^	p-value^2^
**Alcohol consumption**	4.0 (1.8, 6.2)	4.5 (3.0, 6.2)	4.5 (3.0, 7.8)	0.4
**Depression**	3.0 (0.0, 6.2)	5.0 (3.0, 12.0)	11.0 (6.0, 14.0)	**<0.001**
**Anxiety trait**	28 (23, 33)	35 (27, 40)	37 (29, 46)	**0.006**
**Anxiety state**	40 (31, 46)	44 (39, 56)	52 (43, 60)	**<0.001**
**Social support from a close relative**				**0.024**
* No*	10 (21%)	1 (3.1%)	5 (19%)	
* A litle*	19 (40%)	7 (22%)	8 (31%)	
* A lot*	6 (12%)	6 (19%)	7 (27%)	
* Very much*	13 (27%)	18 (56%)	6 (23%)	
**Social support from a friend**				0.8
* No*	4 (8.3%)	2 (6.2%)	1 (3.8%)	
* A litle*	14 (29%)	8 (25%)	9 (35%)	
* A lot*	14 (29%)	7 (22%)	9 (35%)	
* Very much*	16 (33%)	15 (47%)	7 (27%)	
**Coping**	54 (46, 62)	59 (53,64)	61 (54, 66)	**0.029**
**Thought suppression**	46 (36, 56)	52 (43, 59)	62 (57, 66)	**<0.001**

^1^n (%); Median (IQR).

^2^Fisher’s exact test; Kruskal-Wallis rank sum test.

The p-values highlighted in bold are the values that were included in the multivariate logistic regression analysis because they were p < 0.20.

The multivariate analysis identified the difficulties in suppressing intrusive thoughts as the main risk factor for full PTSD (OR = 1.11, 95% CI [1.02-1.21], p < 0.013) (**Table 7**).

**Table 7:** Association between potential post-traumatic predictors and presence of full and partial PTSD in a multivariate logistic regression (N =99).

Characteristic	OR*^1^*	95% CI*^1^*	p-value
Partial PTSD			
Depression	1.01	0.86, 1.18	0.42
Anxiety trait	1.03	0.97, 1.10	0.57
Anxiety state	1.03	0.95, 1.13	0.45
Social support from a close relative	7.31	0.84, 63.9	0.057
Coping	1.03	0.98, 1.09	0.14
Thought suppression	1.00	0.95, 1.05	**0.013**
Full PTSD			
Depression	1.10	0.93, 1.31	0.42
Anxiety trait	1.03	0.96, 1.11	0.57
Anxiety state	0.97	0.88, 1.07	0.45
Social support from a close relative	0.86	0.18, 4.10	0.057
Coping	1.05	0.98, 1.12	0.14
Thought suppression	1.11	1.02, 1.21	**0.013**

^1^OR, Odds Ratio; CI, Confidence Interval.

The p-values highlighted in bold are those that emerged as significant following multivariate logistic regression analysis as they were below p < 0.05.

## 4 Discussion

Faced to the wide variety of PTSD risk factors reported in the literature, we have identified some relevant pre-, peri- and post-traumatic factors associated with the PTSD status 8-18 months after the Paris’ attacks.

The study of pre-traumatic risk factors has shown that, unlike certain findings in existing literature, no socio-demographic data was identified as correlating with the risk of developing PTSD symptoms in this sample, particularly concerning sex, education level, and age at the time of trauma. Their direct correlation with PTSD risk can vary due to multiple factors. These factors often interact in complex ways with other variables, such as the nature of the trauma, available social resources, or cultural differences. Trauma responses and PTSD expression may be influenced by different cultural norms, making generalization of findings challenging. Additionally, the diversity of studied samples and the intricate interplay between sociodemographic factors and other contextual variables can obscure the direct association with PTSD, leading to variability in study conclusions. This absence of association in this study can be attributed to the relatively limited age diversity within the study group, with almost 48% of individuals falling within the 30 to 40 age bracket. Additionally, over 93% of participants possess a bachelor’s degree level of education, encompassing 40% with master’s degree or doctoral degree.

The study confirms that prior exposure to traumatic situations represents a significant risk factor for full PTSD (51). There’s a pressing need to enhance comprehension of the mechanisms involved in this vulnerability post-trauma (52). Notably, no pre-existing factors were identified as predictive of partial PTSD.

Analysis into peri-trauma risk factors has revealed that the intensity of physical reactions during the trauma strongly correlates with the risk of developing PTSD symptoms, both for partial and full PTSD. This observation underscores the significance of peri-traumatic physical reactions in the onset of post-traumatic stress disorder. An abnormal stimulation of the hypothalamic-pituitary system, and autonomic nervous system, the initial points for stress responses during trauma indicated by the intensity of initial physical reactions, could lead to a maladaptive stress response (53–55). This initial response might result in pathological alterations in the mechanisms of traumatic memory formation, as demonstrated by the cortico-limbic system, intricately connected to the hypothalamo-hypophyseal system via the hippocampus and cerebral amygdala, pivotal in managing stress responses, memorization, and forgetting mechanisms (56). These stress-induced changes could also impact the inhibitory system, precipitating the formation of traumatic memory, reflected in intrusions (45). The robust association between physical responses during traumatic events and Post-Traumatic Stress Disorder (PTSD) has garnered substantial support in various studies (13, 14). Two meta-analyses (57, 58), encompassing military, civilian, and police populations, elucidated consistent findings. These analyses underscored the significance of peritraumatic factors as stronger predictors of PTSD in contrast to pretraumatic elements. These intense reactions serve as significant indicators of future post-traumatic symptom severity in certain individuals. However, these studies have revealed that the severity of PTSD can be moderated by factors such as social support, individual coping strategies, or perceived control during the traumatic event (14, 59). These elements play a role in how physical responses manifest into PTSD symptoms evolution, potentially elucidating the variations seen in different study outcomes. The intricate interplay among physical reactions, contextual factors, and individual characteristics emphasizes the necessity of considering these interrelated aspects to comprehensively comprehend their impact on PTSD.

Finally, the examination of post-trauma risk factors has indicated that individuals experiencing full and partial PTSD have difficulties in suppressing intrusive thoughts. This result corroborates functional imaging findings, reinforcing the proposal that the intrusiveness and disrupted control process represents a central aspect in the pathophysiology of PTSD (45, 46). Over the past two decades, evidence has emerged indicating that the prefrontal cortex plays a crucial role in halting retrieval of unwanted memories by targeting memory-related regions, thereby suppressing hippocampal and neocortical activity (60). Compromised control mechanisms and the distress associated with such intrusive thoughts maintain PTSD, but could also constitute risk factors for anxiety and depression (61, 62). Although, our findings suggest an association between depression, anxiety, and an increased risk of PTSD, they do not, however, entirely elucidate this risk when compared to other contributing factors. This suggests that depression and anxiety might co-occur with PTSD rather than directly explain its occurrence.

In this study, we investigated both established risk factors for PTSD from existing literature and novel contributing. Firstly, examining prior trauma exposure is pivotal, as it can heighten vulnerability to subsequent traumatic events. Individuals with a history of trauma may demonstrate increased sensitivity to threatening situations, facing challenges in emotion regulation during stress-inducing circumstances, potentially influencing the onset and severity of PTSD symptoms following new traumatic experiences.

The intensity of physical responses during and after traumatic events is another critical area in PTSD research. Heightened physical reactions, such as intense activation of the autonomic nervous system, may correlate with more severe post-traumatic symptoms. Individuals experiencing extreme physical responses during trauma may exhibit a higher likelihood of enduring PTSD symptoms.

Moreover, difficulties in suppressing intrusive thoughts might exacerbate long-term symptoms as they hinder the healthy processing and silencing of traumatic memories.

Integrating these facets into PTSD research underscores the necessity of comprehending the interplay among trauma history, peri-traumatic reactions, and emotional regulation strategies. This understanding elucidates underlying mechanisms and informs more effective treatment approaches. A holistic approach, considering these interrelated factors, enhances comprehension of individual variability in trauma response and offers pathways for targeted and tailored interventions.

## 5 Implications

Our study explored predictive factors associated with post-traumatic stress disorder (PTSD), specifically within the context of a terrorist attack, providing valuable insights into essential clinical implications. We emphasized that analyzing peri-traumatic physical reactions, coupled with an individual’s traumatic history, could serve as a screening tool during the early phase to gauge the risk of developing PTSD.

In a clinical setting, the use of peritraumatic physical reactions to screen for PTSD could involve systematically assessing immediate physical responses after a traumatic event during post-trauma consultations. Health professionals might utilize standardized questionnaires or scales to gather information about the physical reactions experienced by the patient during or immediately after the trauma. These assessments could be easily used and help identify individuals potentially at risk of developing PTSD, allowing for early and targeted intervention to reduce the risk or severity of the disorder.

Further research is imperative to confirm the role of peri-traumatic reactions in the subsequent onset of PTSD, particularly in the clinical trajectory of PTSD through longitudinal studies.

Additionally, exploring potential connections between the structural and functional alterations associated with peri-traumatic physical symptoms and the operation of central inhibitory control systems (45), alongside amygdalo-hippocampal circuits (56, 63), known to be involved in manifesting classic PTSD symptoms, warrants investigation.

## 6 Strengths and limitations

Our study is founded on a robust methodological framework, albeit with acknowledged limitations discussed below. We employed standardized and scientifically validated measurement instruments, administered by trained professionals, ensuring the reliability of our data. The study population exhibits relative homogeneity concerning proximity to the traumatic event and sociodemographic characteristics. Our design facilitated an early psychopathological analysis of victims.

Furthermore, our study presents a comprehensive evaluation of a population often overlooked in the literature: individuals experiencing PTSD symptoms who do not meet full DSM5 diagnostic criteria, falling under the classification of partial PTSD. This subgroup represents a significant public health concern. Our findings hold potential to refine the identification of these individuals more accurately, enabling prompt intervention for effective treatment.

However, our study does have limitations that should be considered when interpreting the results. The sample size was relatively small, comprising predominantly young and highly educated participants, which limits the generalizability of our findings to the broader population.

The retrospective measurement of peritraumatic physical reactions may lead to reporting and memorization biases, particularly in the case of dissociative reactions. Nevertheless, a prior longitudinal examination of peri-traumatic reactions within this population failed to demonstrate any consequential impact of this memory bias on the findings (64). Furthermore, devising a peri-trauma measure capable of overcoming this limitation proves to be challenging. Finally, our study is based on psycho-pathological data known to be risk factors for PTSD and does not consider physiobiological mechanistic factors. Nonetheless, the risk factors observed in this context of terrorist attacks can provide a framework for further studies to better understand the mechanisms of vulnerability.

## Data availability statement

The original contributions presented in the study are included in the article/supplementary files, further inquiries can be directed to the corresponding author/s.

## Ethics statement

The studies involving humans were approved by Nord Ouest III Personal Protection Committee (France; 12/2016; ID RCB: 2016-A00661-50). The studies were conducted in accordance with the local legislation and institutional requirements. The participants provided their written informed consent to participate in this study.

## Author contributions

BB: Writing – original draft, Writing – review & editing. MT: Writing – original draft, Writing – review & editing. GL: Writing – review & editing. BL: Writing – review & editing. FF: Writing – review & editing. DP: Writing – review & editing. FE: Writing – review & editing. PG: Writing – review & editing. JD: Writing – original draft, Writing – review & editing.
